# Complete coding sequence of the Lumpy skin disease virus collected from cattle in Central Java, Indonesia, 2022

**DOI:** 10.1128/mra.01003-24

**Published:** 2025-03-06

**Authors:** Hendra Wibawa, Lestari, Toan Hong, Zaza Fahmia, Sri Handayani Irianingsih, Herdiyanto Mulyawan, Megaria Ardiani, Rochmadiyanto, Farida Camallia Zenal, Syafrison Idris, Gemma Clark

**Affiliations:** 1 Disease Investigation Centre Wates, Directorate General of Livestock and Animal Health Services, Ministry of Agriculture Indonesia, Yogyakarta, Indonesia; 2 CSIRO - Australian Centre for Disease Preparedness, Geelong, Australia; 3 Directorate of Animal Health, Directorate General of Livestock and Animal Health Services, Ministry of Agriculture Indonesia, Jakarta, Indonesia; 4 FAO - Emergency Centre for Transboundary Animal Diseases to Indonesia, Jakarta, Indonesia; Katholieke Universiteit Leuven, Leuven, Belgium

**Keywords:** Lumpy skin disease virus, coding sequence, whole genome sequencing, cattle

## Abstract

We reported the complete coding sequence of a Lumpy skin disease virus (LSDV) isolated from cattle in Central Java, Indonesia, in 2022. The nucleotide sequence of the virus was most closely related to LSDV strains belonging to clade 2.5, which has been reported in East and Southeast Asia from 2019 to 2021.

## ANNOUNCEMENT

Lumpy Skin Disease (LSD), caused by the Lumpy Skin Disease virus (LSDV), results in significant economic losses to livestock producers. LSDV is a double-strand DNA virus that belongs to the genus *Capripoxvirus* within the family *Poxviridae* and primarily affects cattle. The first LSD outbreak in Indonesia was reported in February 2022 in Riau Province on Sumatera Island (1). By August 2022, the disease had spread to Central Java, the second-largest cattle population in Indonesia. In October 2022, during LSD outbreak in Semarang, Central Java Province, we collected skin nodules (3–4 nodules) from three sick cattle. Samples were transferred in viral transport medium containing antibiotics and stored at −80°C before they were tested. DNA was extracted from pooled skin samples using Purelink Viral RNA/DNA Mini Kit (Invitrogen, Life Technologies) according to the manufacturer’s instructions. A high viral load of LSDV (Ct value = 15.6) was detected by real-time reverse transcription-PCR (rRT-PCR) using SensiFAST Probe Lo-ROX Kit (Bioline, Meridian Bioscience) using primers and probe as previously described (2).

Whole genome sequencing was performed using Oxford NanoPore Technologies (ONT). Genomic DNA was purified using DNA Clean & Concentrator-5 Kit (Zymo Research, USA). About 5 ng of DNA was used for library preparation using the Rapid Barcoding Kit (SQK-RBK004, ONT). The library was sequenced using a MinION SpotON R9.41 FLO-MIN106D flow cell (ONT) for 24 h. Base calling was processed in real-time with MinKNOW Software (v.22.03.05). Base-called reads were concatenated into a single FASTQ file and the adapter sequences were removed using Porechop v0.2.4 (3) with default parameters. NanoStat, a package from NanoPack software suite (4), was used to create a statistical summary from nanopore reads. A total of 273,429 reads were generated with read length N50 of 826. Of these, 247,374 reads were retained after adapter trimming. For genome assembly, trimmed FASTQ files were mapped against the reference genome of LSDV/Thailand/YST/2021 (Accession No. OM033705) using Minimap2 v2.17 (5) and the output (SAM file) was imported to UGENE v50.0 (6) to obtain a consensus sequence. Prokka v.1.14.6 was used for genome annotation (7) using the curated genome of LSDV/Thailand/YST/2021 (8). For phylogenetic analysis, multiple sequence alignment of the obtained genome with 34 virus genomes representing all LSDV clades found in Africa, Europe, the Middle East and Asia (9) was performed using MAFFT v.7.490 (10), followed by maximum likelihood tree reconstruction using IQTREE v.2.2.0 (11).

**Fig 1:**
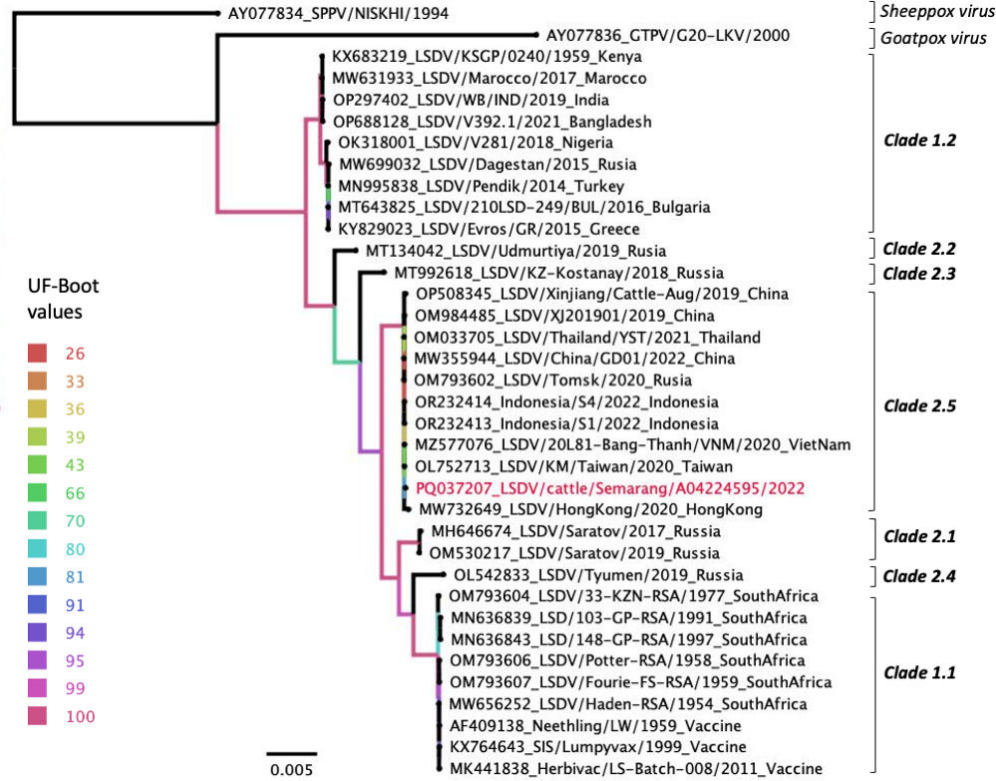
Phylogenetic relationship was inferred by using the maximum-likelihood method based on the complete coding sequence of Lumpy skin disease virus (LSDV) using best-fit K3Pu + F + I + G4 substitution model according to the Bayesian Information Criterion (BIC). The LSDV/ Semarang/A04224595/2022 virus is highlighted (red). The colour of branch indicates the branch support based on 1,000 replicates of ultrafast bootstrap (UF-Boot) approximation (12). Scale bar indicates nucleotide substitutions per site.

The complete coding sequence of LSDV (LSDV/Semarang/A04224595/2022) was successfully obtained from skin nodules of cattle with LSD symptoms during the disease outbreak in Central Java Province in October 2022. The genome has 150,689 bp length with 25.9% GC content with the average sequencing depth was 139.9×. The genome was 99.99% identical (2–4 nucleic acid differences) to 4 LSDV strains detected in China, Taiwan, Vietnam, and Thailand between 2019 and 2021 and to 2 LSDV strains from the index case of LSDV in cattle in Riau Province, Indonesia in 2022 (Table 1). Our phylogenetic tree analysis showed that all the Indonesian LSD viruses are clustered in clade 2.5 (Fig. 1).

**TABLE 1:** Sequence similarity and the number of nucleotide difference in the complete coding sequences of LSDV/Semarang/A04224595/2022 compared to LSDV virus strains isolated from East and Southeast Asia between 2019 and 2022

PQ037207^*a*^ Semarang/ A04224595/2022	OM984485 XJ201901/2019	OL752713 KM/Taiwan/2020	OM033705 Thailand/YST/2021	MZ577076 Bang-Thanh/ VNM/2020	OR232413 Indonesia/S1/2022	OR232414 Indonesia/S4/2022
Homology	99.998%	99.997%	99.999%	99.997%	99.997%	99.998%
Nucleotide difference	3	4	2	4	4	3

^*a*^ GenBank accession number of LSDV virus strains.

## Data Availability

The complete coding sequence of LSDV/Semarang/A04224595/2022 has been deposited in NCBI GenBank under the accession no. PQ037207, and the raw sequence data can be found in the GenBank SRA under BioProject accession no. PRJNA1140204. The version described in this paper is the first version, PQ037207.1.

## References

[1] Sendow I, Meki IK, Dharmayanti N, Hoerudin H, Ratnawati A, Settypalli TBK, Ahmed HO, Nuradji H, Saepulloh M, Adji RS, Fairusya N, Sari F, Anindita K, Cattoli G, Lamien CE. 2024. Molecular characterization of recombinant LSDV isolates from 2022 outbreak in Indonesia through phylogenetic networks and whole-genome SNP-based analysis. BMC Genomics 25:240. doi:10.1186/s12864-024-10169-6 38438878 PMC10913250

[2] Babiuk S, Bowden TR, Parkyn G, Dalman B, Manning L, Neufeld J, Embury-Hyatt C, Copps J, Boyle DB. 2008. Quantification of lumpy skin disease virus following experimental infection in cattle. Transbound Emerg Dis 55:299–307. doi:10.1111/j.1865-1682.2008.01024.x 18503511

[3] Wick RR, Judd LM, Gorrie CL, Holt KE. 2017. Completing bacterial genome assemblies with multiplex MinION sequencing. Microb Genom 3:e000132. doi:10.1099/mgen.0.000132 29177090 PMC5695209

[4] De Coster W, D’Hert S, Schultz DT, Cruts M, Van Broeckhoven C. 2018. NanoPack: visualizing and processing long-read sequencing data. Bioinformatics 34:2666–2669. doi:10.1093/bioinformatics/bty149 29547981 PMC6061794

[5] Li H. 2018. Minimap2: pairwise alignment for nucleotide sequences. Bioinformatics 34:3094–3100. doi:10.1093/bioinformatics/bty191 29750242 PMC6137996

[6] Okonechnikov K, Golosova O, Fursov M, the UGENE team. 2012. Unipro UGENE: a unified bioinformatics toolkit. Bioinformatics 28:1166–1167. doi:10.1093/bioinformatics/bts091 22368248

[7] Seemann T. 2014. Prokka: rapid prokaryotic genome annotation. Bioinformatics 30:2068–2069. doi:10.1093/bioinformatics/btu153 24642063

[8] Suwankitwat N, Songkasupa T, Boonpornprasert P, Sripipattanakul P, Theerawatanasirikul S, Deemagarn T, Suwannaboon M, Arjkumpa O, Buamithup N, Hongsawat A, Jindajang S, Nipaeng N, Aunpomma D, Molee L, Puangjinda K, Lohlamoh W, Nuansrichay B, Narawongsanont R, Arunvipas P, Lekcharoensuk P. 2022. Rapid spread and genetic characterisation of a recently emerged recombinant Lumpy skin disease virus in Thailand. Vet Sci 9:542. doi:10.3390/vetsci9100542 36288155 PMC9609959

[9] Mazloum A, Van Schalkwyk A, Babiuk S, Venter E, Wallace DB, Sprygin A. 2022. Lumpy skin disease: history, current understanding and research gaps in the context of recent geographic expansion. Front Microbiol 14:01–20. doi:10.3389/fmicb.2023.1266759 PMC10652407 38029115

[10] Katoh K, Standley DM. 2013. MAFFT multiple sequence alignment software version 7: improvements in performance and usability. Mol Biol Evol 30:772–780. doi:10.1093/molbev/mst010 23329690 PMC3603318

[11] Nguyen L-T, Schmidt HA, von Haeseler A, Minh BQ. 2015. IQ-TREE: a fast and effective stochastic algorithm for estimating maximum-likelihood phylogenies. Mol Biol Evol 32:268–274. doi:10.1093/molbev/msu300 25371430 PMC4271533

[12] Hoang DT, Chernomor O, von Haeseler A, Minh BQ, Vinh LS. 2018. UFBoot2: improving the ultrafast bootstrap approximation. Mol Biol Evol 35:518–522. doi:10.1093/molbev/msx281 29077904 PMC5850222

